# Auto-Scaling Techniques in Cloud Computing: Issues and Research Directions

**DOI:** 10.3390/s24175551

**Published:** 2024-08-28

**Authors:** Saleha Alharthi, Afra Alshamsi, Anoud Alseiari, Abdulmalik Alwarafy

**Affiliations:** Department of Computer and Network Engineering, College of Information Technology, United Arab Emirates University, Al Ain 15551, United Arab Emirates; 700042190@uaeu.ac.ae (S.A.); 201410170@uaeu.ac.ae (A.A.); 201515924@uaeu.ac.ae (A.A.)

**Keywords:** auto-scaling, cloud computing, workload forecasting, machine learning

## Abstract

In the dynamic world of cloud computing, auto-scaling stands as a beacon of efficiency, dynamically aligning resources with fluctuating demands. This paper presents a comprehensive review of auto-scaling techniques, highlighting significant advancements and persisting challenges in the field. First, we overview the fundamental principles and mechanisms of auto-scaling, including its role in improving cost efficiency, performance, and energy consumption in cloud services. We then discuss various strategies employed in auto-scaling, ranging from threshold-based rules and queuing theory to sophisticated machine learning and time series analysis approaches. After that, we explore the critical issues in auto-scaling practices and review several studies that demonstrate how these challenges can be addressed. We then conclude by offering insights into several promising research directions, emphasizing the development of predictive scaling mechanisms and the integration of advanced machine learning techniques to achieve more effective and efficient auto-scaling solutions.

## 1. Introduction

Cloud computing enables users to access and utilize a range of computing resources, including networks, servers, storage, and applications through the internet [1]. According to the National Institute of Standards and Technology, it is a system where you can easily access and use these resources whenever you need them without much hassle or needing to interact a lot with the service provider [2]. Cloud computing has emerged as a critical tool and is being used across various sectors, such as e-learning [3], healthcare [4], finance and banking [5], manufacturing [6], and telecommunications [7].

In settings where data-intensive tasks are prevalent, crucial cloud computing features like elasticity, resource allocation, and pooling, play significant roles. Scalability, a key component of cloud computing, empowers organizations to modify their resource use to align with their needs, offering not only cost benefits but also crucial enhancements in the performance of cloud-based applications [2]. The critical nature of scalability becomes particularly apparent in the realm of large-scale graph processing, which finds application in media, online gaming [8], and the internet of things (IoT) [9]. Conventional tools for graph processing often falter with large-scale graphs, facing difficulties in balancing scalability and cost-efficiency. [10]. Researchers in [11], discussed issues related to large-scale optimization in elastic cloud computing. Auto-scaling, which involves dynamically increasing or decreasing resources based on specific needs and strategies, addresses this challenge by providing the flexibility to adapt.

In this review paper, we explore the concept of auto-scaling in cloud computing, a critical mechanism that adjusts resources dynamically based on current demands. Section 2 covers some existing works related to auto-scaling techniques. Section 3 lays the foundation by introducing the fundamentals of auto-scaling, particularly emphasizing its relevance in the cloud computing context. Following that, Section 4 delves into the algorithms predominantly employed in auto-scaling within cloud environments, offering a detailed examination of their operations and applications. In Section 5, we transition to practical insights by showcasing the real-world applications of auto-scaling, illustrating how this technology is implemented in various scenarios. Section 6 addresses the challenges associated with auto-scaling in cloud computing. Section 7 presents potential prospects in this evolving field. Finally, we conclude the work in Section 8. Table 1 provides a list of the acronyms used in this paper and their definitions.

## 2. Related Work

Existing review papers have been published [12,13,14,15,16,17,18,19,20,21], and they have provided valuable insights. However, with recent updates in the cloud computing environment, new research areas have emerged. It is necessary to explore the current key challenges to propose future research directions in this field. Therefore, this review covers auto-scaling techniques and discusses the current challenges. Furthermore, we discuss the current applications and domains of auto-scaling techniques. Table 2 illustrates the existing surveys and compares them with our work.

To the best of our knowledge, no previous review paper has focused on all types of auto-scaling techniques used in cloud environments. In this paper, we highlight different types of auto-scaling techniques such as reactive methods (threshold rules, queuing theory) and proactive methods (reinforcement learning, fuzzy learning, machine learning, and time series analysis).

## 3. Fundamentals of Auto-Scaling

This section explores the fundamental concepts of cloud computing, auto-scaling, horizontal scaling, and vertical scaling. These concepts lay the foundation for the remainder of the review paper, it ensures that readers have a clear insight of fundamental concepts before delving into detailed analyses.

**Figure 1:**
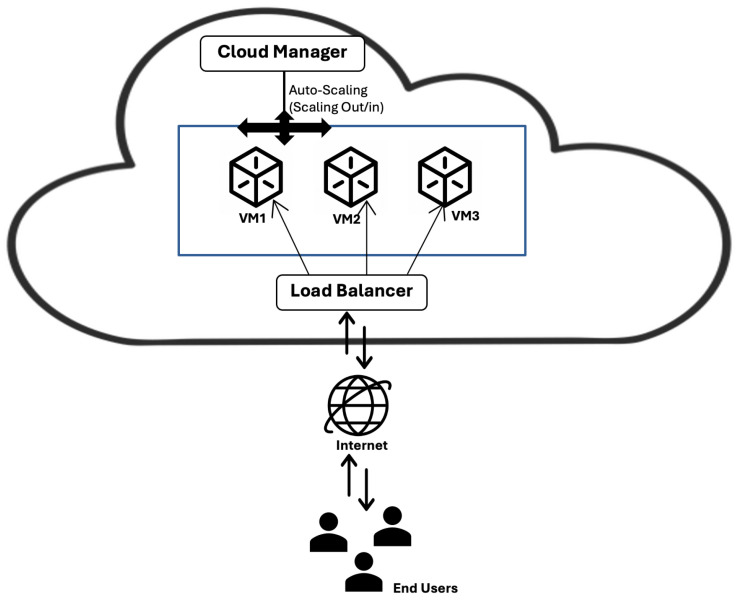
Auto-scaling in cloud computing.

Cloud computing has swiftly become an essential part of daily life and is increasingly favored by Internet users [22]. Users of cloud services pay only for the resources they use, following a pay-per-use model. However, achieving a balance between cost-effectiveness and performance requires precision. As shown in Figure 1 the architecture of cloud computing consists of various components. The aim is to deliver top-notch cloud services at minimal costs while ensuring prompt response to requests and system stability. Scalability, a pivotal characteristic of cloud computing, addresses this challenge by enabling flexibility and efficiency for users. To clarify the meaning of auto-scaling, auto-scaling refers to the ability to scale in and out by adding or removing resources depending on the requirements [7].

Auto-scaling in cloud computing involves restructuring the allocation of resources in hardware and software with specific requirements [23]. Cloud computing provides several features such as scalability, security, efficiency, and performance. It has been deployed in different industries [7]. Cloud computing is widely adopted due to its flexibility and scalability, which are essential for handling big data. However, optimizing cloud resources for auto-scaling to manage big data efficiently remains a challenge [24]. In a cloud environment, reactive auto-scaling relies on a set of thresholds, and it cannot predict a system’s future behavior. For example, if the value of a particular parameter such as average CPU usage exceeds a threshold, additional CPU capacity will be added to the system [23].

Cloud-based applications can automatically adjust their resource usage, increasing or decreasing as required to meet the demands of the application. The key features of auto-scaling include the ability to add resources during peak demand (scaling out) and to remove unused resources to minimize costs when demand decreases (scaling in). Rules can be set for scaling actions, and unhealthy instances can be detected automatically and replaced. Auto-scaling is often discussed in the context of resource provisioning, scalability, and elasticity, which are related but distinct concepts [25].

Resource provisioning enables a system to scale resources dynamically based on workload changes [26], improving scalability and performance. Scalability refers to a system’s ability to handle increasing workloads by adding hardware resources [27], either horizontally (scaling out) by adding nodes or vertically (scaling up) by increasing resources in existing nodes. Scalability is a prerequisite for elasticity, which involves adapting to workload changes by provisioning and deprovisioning resources autonomically to closely match demand. Elasticity encompasses how quickly a system can respond to workload fluctuations, ensuring that the available resources closely align with the current demand. Auto-scaling techniques facilitate elasticity by enabling automatic resource adjustments [25].

Auto-scaling solutions are classified based on horizontal and vertical scaling methods. Horizontal scaling is when the virtual machines are added or removed to match the requirements of the current workload. Vertical scaling offers tools for removing or adding resources (e.g., RAM, CPU, and disk space) from the VM instances [28].

**Figure 2:**
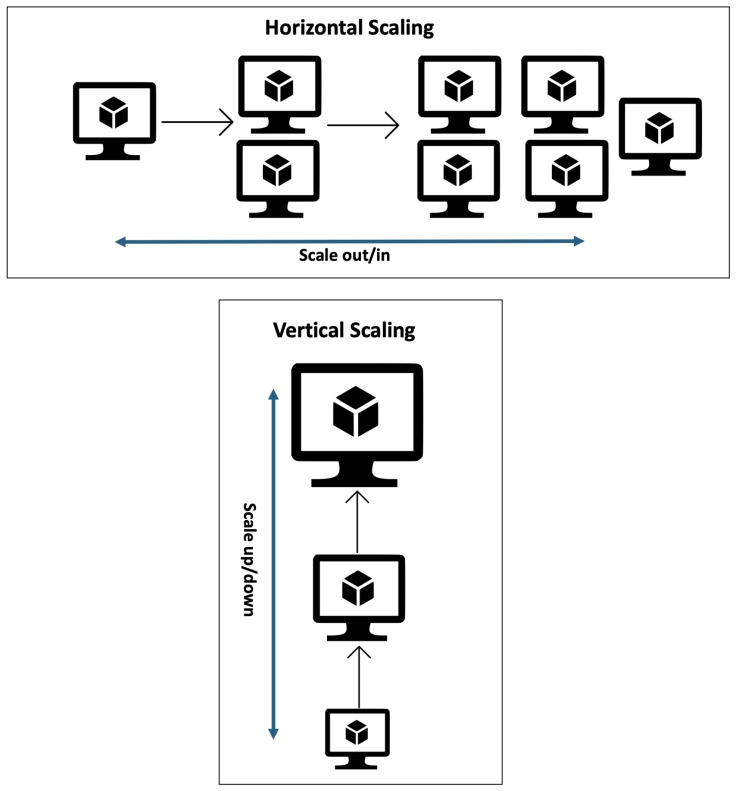
Horizontal vs. vertical scaling concepts.

Horizontal scaling increases the number of existing resources by adding extra machines. Vertical scaling means that when adding new machines to a system, large resources may need to be broken down into smaller types to reduce costs. Removing or downward machines from a pool of resources of the same type is referred to as homogeneous, and machines of different types are called heterogeneous [10]. Figure 2 shows the difference between the horizontal and vertical scaling methods.

The paper [29], extensively reviews auto-scaling and load balancing technologies in cloud computing, highlighting their pivotal role in efficiently managing data and applications through centralized resources. Focusing on infrastructure as a service (IaaS), it emphasizes dynamic scaling and load distribution techniques crucial for optimizing resource utilization and performance across varied workloads. A substantial section of the paper (illustrated in Table 3) delves into a comparative analysis of how major cloud providers like amazon web services (AWS) and Microsoft azure implement these technologies. AWS utilizes auto-scaling within its EC2 platform to adjust instance numbers dynamically based on application demand, using load balancers to distribute traffic effectively. In contrast, Microsoft Azure uses a platform-as-a-service model that depends on external tools such as Paraleap for auto-scaling, showcasing diverse approaches in the deployment and management of these critical cloud computing features [29].

## 4. Auto-Scaling Techniques

This section presents a detailed discussion of the reactive and proactive auto-scaling methods. Auto-scaling is either reactive or proactive. Reactive methods in auto-scaling refer to the allocation and deallocation of resources only when necessary. Furthermore, reactive auto-scaling is the most commonly used in the market. However, proactive auto-scaling attempts to scale resources while considering future workload patterns. A proactive mechanism can handle sudden workload fluctuations by estimating the required number of resources, whereas a reactive mechanism cannot handle such fluctuations [28,30]. A proactive auto-scaling mechanism [31], can use past data on workload patterns. Hence, by using a predictive technique on these data, they can estimate the future resources that are needed for the future workload. In addition, the performance of proactive auto-scaling depends on the accuracy of the workload prediction method. The integration of both methods can improve a system’s throughput as discussed in [32], they introduced a hybrid auto-scaling approach that utilizes reactive and proactive methods.

### 4.1. Reactive Methods

#### 4.1.1. Threshold Rules

Threshold-based rules are popular due to their simplicity and ease of deployment in cloud infrastructure. Cloud service providers, such as Amazon and Azure, offer rule-based solutions that depend on user-defined rules and scaling actions when specific conditions are met such as when CPU usage exceeds a specific threshold [33]. The threshold-based rule technique is related to planning; however, defining rules is the most difficult part because it requires user input concerning various quality of service (QoS) metrics. The common metrics used in the threshold evaluation were performance, CPU load time, and application response time. The parameters are obtained from monitoring tools and real-time data that can be used to perform actions based on rules [33]. This technique follows a reactive approach as it responds to the collected data. Rule-based techniques can be easily implemented in specific applications with predictable workloads. However, if the application has an unpredictable pattern, this technique is not the right choice. In that case, it is better to use other techniques. Using the threshold strategy has several advantages; for example, it enhances system performance to avoid VM start and shutdown time. In [34], the authors discussed prediction frameworks related to VM and conducted several experiments indicating that prediction frameworks can improve the quality of cloud services. Furthermore, it reduces costs and improves energy consumption. In [35], the authors proposed an energy-efficient threshold to reduce energy consumption; thus, they used threshold values to trigger task migration between virtual machines (VMs). This technique significantly reduced the Makespan and increased resource utilization.

#### 4.1.2. Queuing Theory

Queuing theory is utilized in the analysis stage of auto-scaling and evaluates the performance parameters. It helps understand a system’s performance based on metrics such as response time for requests, queue length, and service rate. One drawback of queuing theory is that it is intended for stationary natural systems; thus, the dynamics of clouds are not addressed. To address this issue, researchers can combine queuing theory with other techniques, such as control theory and threshold-based policies. In [36], researchers presented optimization methods based on a combination of queuing theory and the Markov decision process (MDP). This study benefits service providers by optimizing their financial costs because it consists of a set of fast load-based threshold techniques. However, the scope of their paper is only focused on queue thresholds that determine when to activate or deactivate the number of VMs. In contrast, the authors of [37] used the queuing theory model in the context of VM migration to provide performance metrics for estimations and improve resource utilization by supporting decision-making processes. The queuing theory-based model helps increase resource utilization, improve QoS, and enhance resource scaling decisions. Furthermore, it can improve energy efficiency by identifying the most energy-efficient migration trends and server utilization scenarios.

### 4.2. Proactive Methods

#### 4.2.1. Reinforcement Learning

RL is a commonly used machine learning paradigm that involves agent interaction with the environment. In [16,17], the authors provided valuable insights into the application of RL to address decision-making issues in cloud computing. They mentioned that the auto-scaler acts as an agent that performs actions based on the environmental state. Initially, the agent does not know what actions to perform; as time passes, the agent should explore the appropriate decision to perform certain actions. Therefore, RL allows agents to interact with the environment, which allows them to discover and learn how to make decisions and increase the cumulative rewards. In [38], the researchers proposed the RL model as a solution, and they focused on predicting the usage of automation applications such as XR/VR. The RL agent is trained to define the workload and predict the usage of services. The results of the RL-based model were compared to those of standard reactive techniques. They found that the RL agent outperformed the reactive scalers in terms of obtaining higher reward and utilization levels in microservices. This means the agent can learn how to scale microservices in advance. Although this research paper relies on simulations alone, the proposed approach may not address the challenges of an actual system. However, the authors of [39] conducted real-world experiments on their proposed auto-scaling microservice based on RL, which was used to test and validate the module and identify the auto-scaling threshold value. The study in [40], proposed a new auto-scaling policy using RL.

#### 4.2.2. Fuzzy Learning

Fuzzy learning, also known as fuzzy control or fuzzy logic, is a rule-based auto-scaling technique that involves a set of predefined if–else rules. It allows users to use high, medium, and low linguistic terms and can be used to define appropriate scaling decisions based on fuzzy rules. These rules determine the correlations between input variables, such as response time, CPU utilization, and increasing or decreasing resources. This can improve system performance and efficiency by enabling resource allocation based on current workload rules. In [41], the authors used a fuzzy logic control system to prioritize processes based on the system workload. They used input parameters such as processing time, workload, and process age. Similarly, in [42], a fuzzy logic technique was used to define the target layers for offloading considering the resource heterogeneity of different input parameters such as task size, latency, and network bandwidth. A fuzzy learning technique can be implemented to make decisions about task offloading, as discussed in [43]. Fuzzy logic is employed as a task scheduler to define the target processing layer for an IoT device’s heterogeneous task. These studies proposed innovative solutions using fuzzy techniques. However, incorporating machine learning and fuzzy learning techniques can enhance scalability in cloud computing with minimal reliance on expert-defined fuzzy learning rules.

#### 4.2.3. Machine Learning (ML)

**Figure 3:**
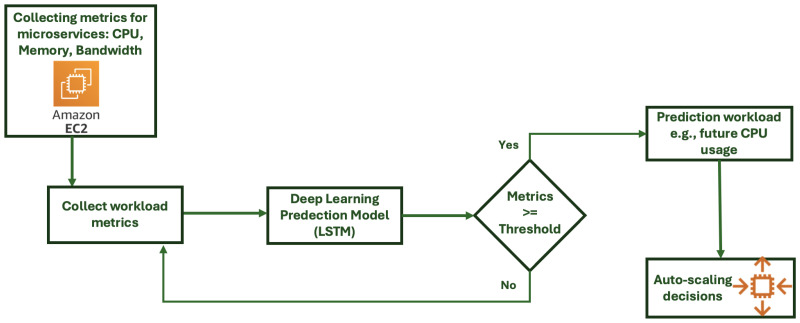
Real-time auto-scaling system based on deep learning.

This technique is widely used to enhance auto-scaling in cloud computing and predict future workloads. Furthermore, it makes accurate predictions about future resource requirements because it evaluates historical data and monitors real-time metrics, such as memory usage, CPU utilization, and network traffic. ML algorithms use these metrics to scale resources up or down based on specific demands. Machine learning involves various techniques, such as neural networks, support vector machines, and linear regression. One of the existing models [30] has shown that predicted data are very close to the real data. Figure 3 illustrates a real-time auto-scaling system based on deep learning that can forecast future workloads, such as the value of CPU, bandwidth, and memory. Furthermore, it demonstrates the process of collecting metrics from various microservices, such as AWS EC2 instances. However, the model forecasts the workload, and based on these predictions, the system decides to scale resources up or down automatically. To address the issue of estimating the right threshold value for microservices, the authors of [39] proposed an intelligent autonomous auto-scaling system. They combined existing machine learning techniques with RL, and the results indicated that the microservice response time was improved by up to 20% compared to the default auto-scaling model. Another proposal introduced in [44] also addressed the challenges of auto-scaling in microservices in terms of performance prediction and resource allocation. They applied particle swarm optimization to a deep neural network (DNN) to realize optimal resource allocation. The results indicated that the proposed technique improved performance while minimizing the total cost of CPU by 30% to 50% compared to the baseline. Prior work [45] discussed the importance of incorporating ML into AL-driven DevOps, which can lead to significant improvements in cloud computing infrastructure. In [46], the authors developed an ML approach to improve the efficiency and performance of cloud computing resources. In [47], the authors described ML solutions in large-scale systems. In [48], the authors proposed a solution for proactive mission-critical services (MCS) and described how the metrics provided by MCS can predict future requirements to realize service scalability actions.

#### 4.2.4. Time Series Analysis

Time series analysis is a popular technique that has been applied in various fields, such as engineering, finance, and bioinformatics. Generally, it represents the variation in measurement over time. This method is employed in auto-scaling to forecast future workloads. By exploiting this technique, users can dynamically scale up or down their resources over time while ensuring optimal resource usage. As represented in [49], the resource utilization was reduced by up to 71%, through the integration of time series analysis with Daedalus, which is a self-adaptive manager for auto-scaling. A time series forecasting technique [30] collects historical data and uses statistical approaches to determine trends. These historical data were collected at regular intervals for example each five or two minutes. Different time series forecasting models are trained to use historical data and then use them to forecast future resource demand. In [50], researchers used time series analysis to define citizen-reported urban issues and enable forecasting of future urban challenges. They provided insights into employing this technique which is a powerful statistical method to analyze sequential data points gathered over time. Practical application of time series analysis demonstrated in [51], they compared three AutoML tools and selected three types of time series datasets: weather, Bitcoin, and COVID-19. The main objective of using these tools is to reduce reliance on computer science expertise and improve the application of ML tools for time series analysis and predictions. In [52], researchers developed an ML approach called IoTArchML that continuously monitors QoS parameters and forecasts QoS metrics based on historical data.

## 5. Real-World Applications

Auto-scaling techniques have become indispensable in various sectors, showcasing their adaptability and critical role in resource optimization and performance enhancement across diverse computing environments. This section reviews real-world applications of auto-scaling, illustrating its effectiveness in ensuring efficient operations and resource utilization in smart city infrastructure [23], social media analytics [24], telecommunications [7], and healthcare system [53].

### 5.1. Smart City Platforms

In the realm of smart city platforms, the dynamism of computing services is crucial due to the highly variable workloads stemming from multiple user interactions. Researchers in [23] have emphasized the necessity for auto-scaling in smart city infrastructure to maintain service efficiency and adaptability. The study demonstrates how a decision-making mechanism for auto-scaling can be tailored to user specifications within such environments, revealing that settings like update history and heat thresholds are pivotal in influencing auto-scaling behavior. This underscores the technique’s potential to optimize resource allocation in response to fluctuating demands within smart city systems.

### 5.2. Sentiment Analysis on Twitter Data

Another compelling application was presented in the paper [24], where a cost-effective auto-scaling framework was employed for the sentiment analysis of Twitter data from various universities. The proposed framework adjusts computing resources within a Hadoop cluster, catering to the real-time processing needs of vast Twitter datasets. By adeptly scaling resources to match data loads, the framework not only optimizes utilization but also curtails unnecessary expenditures, showcasing an efficient data processing paradigm that yields valuable insights into public sentiment, crucial for academic institutions to gauge their social media engagement and public perception.

### 5.3. Telecommunications (Telco) Cloud Infrastructure

The telecommunications sector also benefits from auto-scaling, especially when integrating containerized and virtualized network functions. As discussed in [7], an auto-scaling architecture tailored for the Telco cloud employs algorithms that are conducive to both containerized network functions (CNFs) and virtualized network functions (VNFs). This architecture features an elastic auto-scaling system, orchestrating management, and load balancing to adeptly handle application loads across various VMs and pods. Such a system not only ensures resource efficiency but also facilitates cost reduction and enhanced service reliability in telecommunications infrastructures.

**Figure 4:**
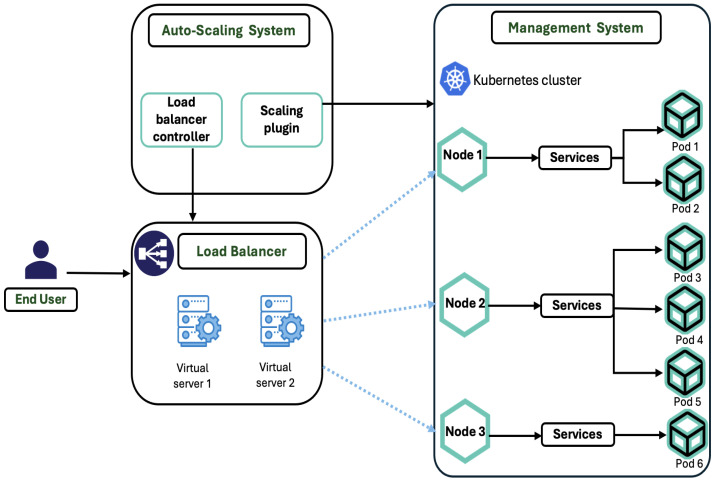
Auto-scaling system architecture.

Figure 4 shows an elastic auto-scaling architecture similar to the one proposed in [7]; it is deployed in the Telco cloud and consists of three main components: an auto-scaling system, orchestration and management, and load balancer.

The role of the load balancer is to balance the Telco application load by balancing requests between different pods and VMs that come from different destinations in the cluster. The management system is used to manage the VMs and pods in Kubernetes and gather the resources utilized and other parameters of each pod and VM running on the cloud. The auto-scaling system includes a load balancer controller, address collector, and scaling plugin. It can expand the number of pods and VMs horizontally based on the workload of the virtual cluster. Once the virtual cluster consumes the most resources and exceeds the defined threshold, the auto-scaling system creates a new VM that executes the same application and load balancer to balance the requests between the VMs.

### 5.4. Application of Auto-Scaling in Healthcare

**Figure 5:**
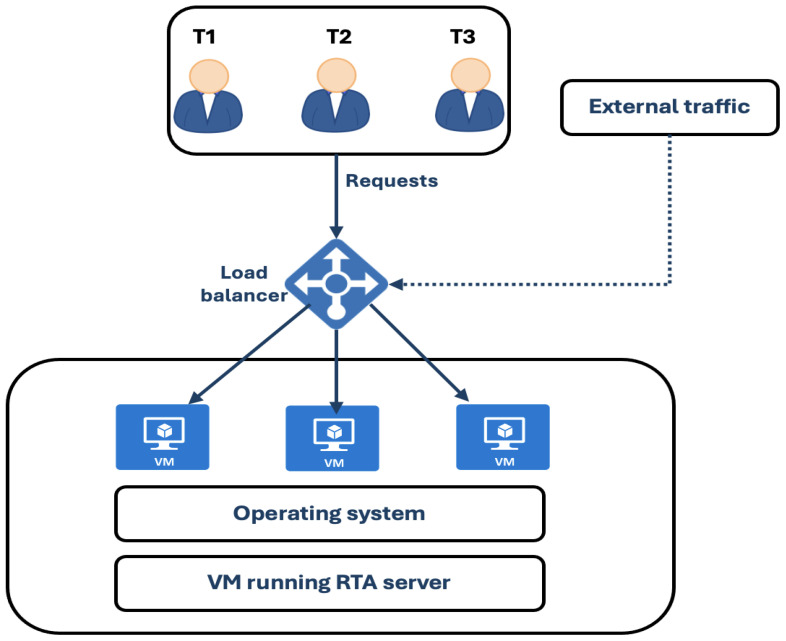
Physical machine architecture.

The auto-scaling mechanism described in [53], has significant practical applications in real-world healthcare settings, particularly in enhancing the capabilities of cloud-based medical monitoring systems. By implementing this proactive auto-scaling solution, healthcare providers can ensure that their cloud infrastructures dynamically adjust to fluctuating data volumes from various medical devices, such as electrocardiograms or continuous glucose monitors. For example, in a scenario where a patient’s condition deteriorates, the system can scale up resources predictively to handle the increased data transmission from the monitoring devices, ensuring that all critical data are processed in real time. This approach allows timely medical intervention based on the most recent health data. In essence, the application of this auto-scaling mechanism in real healthcare settings contributes to a more robust, responsive, and efficient medical monitoring system, reducing latency and enhancing the accuracy of patient care by leveraging cloud computing’s flexible resource management. As shown in Figure 5, the architecture demonstrates how multiple VMs are utilized within the cloud to support such dynamic resource allocation effectively.

## 6. Challenges and Possible Solutions

In the rapidly evolving cloud computing domain, the ability to dynamically adjust computational resources in response to fluctuating workloads, known as auto-scaling, represents a pivotal innovation. This functionality is crucial for optimizing resource utilization, maintaining service level agreements (SLAs), and ensuring high QoS for end users. Despite its benefits, auto-scaling presents a unique set of challenges, primarily due to the variable nature of cloud workloads, which demands a system that can anticipate and react to changes efficiently.

Auto-scaling in cloud computing presents a multitude of challenges that can impact its effectiveness and efficiency. A primary concern is the accuracy of resource prediction, which is crucial for provisioning the appropriate amount of resources dynamically. Incorrect predictions can lead to significant resource wastage or performance bottlenecks, affecting operational costs and application reliability. Additionally, applications in sectors like healthcare and finance may experience sudden spikes in demand, which auto-scaling systems must manage in real-time to prevent performance degradation. Integrating auto-scaling solutions with existing IT infrastructures, especially legacy systems, introduces further complexity. This integration often results in deployment challenges and security vulnerabilities. Moreover, the auto-scaling operations themselves can consume substantial resources, potentially detracting from the overall performance of the cloud environment [54]. To address these challenges, several solutions can be implemented to enhance the functionality and reliability of auto-scaling in cloud environments. Improving the accuracy of predictive algorithms through advanced machine learning techniques can help in dynamically adjusting resources more accurately, based on historical data and real-time analytics. Implementing hybrid scaling strategies that combine proactive and reactive methods can effectively manage rapid demand fluctuations. Developing cost-aware auto-scaling algorithms that incorporate financial metrics into the scaling decision process can optimize operational costs while maintaining performance. To reduce the complexity of implementing auto-scaling in existing systems, standardizing auto-scaling interfaces and protocols is crucial along with ensuring that new systems are compatible with older technologies through comprehensive testing. Additionally, optimizing the efficiency of scaling algorithms and minimizing the resource footprint of auto-scaling processes can mitigate the performance overheads associated with auto-scaling operations. For legacy systems, developing middleware that can bridge the gap between scalable cloud infrastructure and non-scalable legacy systems may provide a viable solution [54].

This paper delves into details of the various challenges associated with auto-scaling in cloud environments, exploring the implications of workload variability and the need for predictive scaling mechanisms to prevent resource wastage and service degradation. We summarized recent innovative solutions across different cloud computing paradigms, including Hadoop-based big data processing, Telco cloud services, graph processing, and general cloud applications.

### 6.1. Energy Efficiency

The energy efficiency challenge in Apache Hadoop, as discussed in [2], stems from its significant energy consumption in data centers. The architecture of Hadoop’s distributed file system (HDFS) complicates the ability to scale down resources efficiently, leading to servers running idle and consuming energy without active use. This inefficiency not only increases operational costs but also impacts the environmental footprint of data processing. The challenge is further exacerbated by the necessity to maintain data availability, preventing the straightforward deactivation of idle nodes, thus hindering the optimization of energy usage in Hadoop deployments.

To address this challenge, the document proposes an innovative solution that involves separating HDFS nodes from MapReduce computing nodes, enabling more effective auto-scaling. This separation allows for the dynamic scaling of computation nodes independent of the data nodes, thereby maintaining data availability while optimizing resource utilization. The introduction of a cluster monitor and auto scaler component enables the system to make informed decisions about when to scale up or down based on real-time metrics like CPU and RAM utilization, and job task status. This approach not only enhances energy efficiency by shutting down unnecessary compute nodes during low workload periods but also ensures the system’s responsiveness and capacity to handle bursts of computational demand, paving the way for a more energy-efficient and cost-effective Hadoop deployment.

### 6.2. Dynamic Nature of Telco Services

In [7], the authors presented challenges and solutions for implementing elastic auto-scaling in Telco cloud environments. The primary challenge is the dynamic nature of Telco services, especially with the advent of 5G technology, which necessitates rapid and reliable service provisioning. This challenge is compounded by the coexistence of VNFs and CNFs in the Telco cloud, necessitating an auto-scaling architecture that can adapt to both. The challenge is to manage resource allocation dynamically to address varying user demands without under-provisioning (which can lead to service degradation) or over-provisioning (which is costly and inefficient).

To address these challenges, the paper [7] proposed an elastic auto-scaling architecture tailored for the Telco cloud, accommodating both VNFs and CNFs. This architecture includes a load balancer, a management and orchestration system, and an auto-scaling system with two specialized algorithms. The architecture is designed to automatically scale services in or out based on real-time demands, enhancing resource utilization and efficiency. By integrating with existing Telco cloud infrastructures, this solution aims to ensure that services can dynamically adapt to varying loads, thereby optimizing resource use, reducing costs, and maintaining service quality regardless of demand fluctuations.

### 6.3. Optimizing Big Data Processing with Auto-Scaling in the Cloud

The primary challenge identified in [24] concerns the efficient processing of big data within cloud computing environments, where the main issue lies in the dynamic adjustment of computing resources to handle varying data workloads efficiently. Traditional fixed resource configurations, designed to manage peak loads, result in underutilization and wastage during periods of lower demand, leading to inefficiency and escalated costs.

In response to this challenge, the authors [24] find a solution through a cost-effective auto-scaling (CEAS) framework specifically developed for the AWS cloud. This framework intelligently auto-scales the virtual nodes in a Hadoop cluster, aligning resource allocation with the fluctuating real-time data workloads. By dynamically scaling resources up or down based on actual need, the CEAS framework optimizes resource usage, thereby enhancing operational efficiency and reducing the financial burden associated with big data processing in the cloud.

### 6.4. Cost-Efficient Graph Processing in Clouds

The public cloud providers offer a pay-as-you-go model that allows users to pay only for utilized resources. However, there are issues related to using such a model, for example, infrastructure virtualization overhead, latency due to VM placement, and a controlled environment. To address these issues, Ref. [10] developed a new resource-based auto-scaling algorithm that enables the partitioning of graphs through available VMs based on VM type. The proposed solution [10] is a new auto-scaling algorithm, and it interfaces it into the iGraph framework to minimize the cost of graph processing in the cloud environment. Further, it introduces a new feature-based dynamic approach that distributes graph partitions effectively on heterogeneous resources. The auto-scaling algorithm can dynamically change the types and number of virtual machines based on the required capacity to process the rest of the graph.

### 6.5. Enhancing Cloud Auto-Scaling with Machine Learning-Based Workload Prediction

The main challenge in cloud computing, as presented in [28], revolves around handling fluctuating workloads in VMs to maintain optimal resource allocation. Cloud services must adapt to varying workload patterns by autonomously scaling resources up or down to ensure adherence to SLAs and maintain QoS. The inherent issue is the dynamic nature of cloud workloads, which necessitates predictive scaling to avoid both over-allocation and under-allocation of resources, which respectively lead to resource wastage and potential degradation in service response times.

To address this challenge, the authors [28], propose a proactive auto-scaling solution using a ML based model, specifically utilizing a SVM for multi-class classification. This model predicts future workload trends by learning from historical data, enabling the cloud system to prepare and adjust the number of VMs in advance to meet the predicted demands. This predictive approach aims to optimize resource utilization, ensuring that the cloud infrastructure dynamically aligns with user demand, thereby enhancing operational efficiency and reducing unnecessary costs. The effectiveness of the proposed model is demonstrated using a real cloud dataset, showcasing its potential to accurately predict workload changes and facilitate more informed auto-scaling decisions in cloud environments.

## 7. Research Directions

This section thoroughly investigates crucial future directions for auto-scaling in cloud computing infrastructure. Based on our survey, we outlined some potential research directions for future improvements, as discussed below.

### 7.1. Energy Efficiency

Recently, several studies have focused more on cost optimization and the QoS in auto-scaling. However, optimizing energy is another promising research field that requires further investigation. Energy consumption is a major concern in auto-scaling and some of the existing work tried to minimize energy utilization. In [35], researchers proposed an energy-efficient threshold to reduce energy consumption. Also, in [2], this approach enhanced energy efficiency by shutting down unnecessary compute nodes during low workload periods. Another important research direction that needs further exploration is applying auto-scaling techniques to reduce energy consumption. As previously mentioned in [37], the queuing theory-based model helps increase resource utilization and improves energy efficiency by determining the most energy-efficient migration trends and server utilization scenarios. Few studies have incorporated renewable energy sources, such as solar panels, to provide power to data centers. These solutions are critical future research directions that require additional investigation. The objective of adopting renewable energy is to boost environmental sustainability by minimizing the dependence of data centers on non-renewable energy sources during auto-scaling decisions. For sustainable energy solutions, the authors of [55] encouraged researchers to explore more the combination of hydropower dams with floating photovoltaic energy. In [56], the authors developed a multi-agent deep reinforcement learning framework to address the challenges of renewable energy in data centers. They successfully combined green and brown energy sources. However, the framework was demonstrated through simulations; thus, adopting such a system in practical cloud computing applications requires further investigation. In [57], researchers investigated the best option in terms of power saving in both cloud computing and edge computing.

### 7.2. Workload Prediction

Recent studies have demonstrated that proactive auto-scaling techniques can be incorporated with ML algorithms to enhance workload prediction. One promising research direction is refining prediction accuracy in SVM, deep learning, and time series analysis algorithms. These enhancements improve system performance and provide highly accurate workload predictions. As explained in [28], they used ML and SVM to predict future workload trends by learning from historical data, enabling the cloud system to prepare and adjust the number of VMs in advance to meet predicted demands. Another model, in [30], developed a real-time auto-scaling system based on deep learning and it indicates that predicted data are very close to the real data.

### 7.3. Cost Optimization

Spot instances have emerged recently as a promising solution introduced by AWS that minimizes the cost of resources compared to on-demand resources. Another promising research direction that requires further investigation is the CEAS framework developed by AWS, as discussed in [24]. This framework optimizes resource usage, thereby enhancing operational efficiency and reducing the financial burden associated with big data processing in the cloud. In addition, in [10], they developed a framework to minimize the cost of graph processing in a cloud environment, which requires further exploration. Cost-aware auto-scaling techniques are a critical area of future research. Several optimization techniques, such as queuing theory and reinforcement learning, can be explored to provide cost-effective auto-scaling strategies. In a different perspective on RL, as discussed in [58], the authors utilized the DeepScale algorithm to auto-scale containerized applications and reduce costs. Also, in [59], researchers employed multiple ML techniques to reduce resource costs.

### 7.4. Combining Vertical and Horizontal Scaling

Although several studies have applied hybrid scaling approaches, they have not addressed the challenges related to the integration of vertical and horizontal scaling, such as resource allocation, monitoring, decision-making, and load balancing. Therefore, further investigation in these areas could provide viable solutions to enhance hybrid scaling approaches. Applying hybrid scaling is a promising avenue for research and provides more flexible solutions to enhance resource usage, as discussed in [25]. The authors showed the differences between Knative hybrid auto-scaling and default horizontal auto-scaling. Results indicated that their system outperformed the existing default Knative horizontal auto-scaling. In [60], they conducted a comparison of two auto-scaling approaches under Kubernetes. While in [61], researchers proposed a hybrid auto-scaling framework specifically for containerized in Kubernetes to enhance resource utilization. Furthermore, for future improvements, the authors suggest determining a mechanism for selecting an appropriate monitoring interval length.

### 7.5. Real-Time and Proactive Auto-Scaling

Unlike traditional auto-scaling, which has latency issues, real-time and proactive auto-scaling is a new paradigm that researchers should continue to explore. Some studies have implemented real-time and proactive methods that can proactively modify resources in advance and accurately predict workload changes. Implementing these algorithms has several benefits. For example, latency issues related to traditional auto-scaling can be eliminated, thereby ensuring a faster response time and more efficient system performance. Another approach is predicting future workloads to optimize resource allocation, which leads to saving more cost. The proposed system in [62] was able to predict future workload with multiple metrics to scale edge computing resources up and down. Overall, these techniques minimize response times and enhance system performance. As previously mentioned in [30], they developed a real-time auto-scaling system based on deep learning, and their results indicated that their predicted data are very close to the real data. However, gaps remain in these studies and require further evaluation. Hence, real-time and proactive auto-scaling is one of the most important research directions in auto-scaling and cloud computing infrastructure.

### 7.6. Heterogeneous Resource Provisioning

Heterogeneous resource provisioning is another important research area that requires further exploration. There are diverse types of resources in cloud computing such as VMs, containers, storage, and serverless functions. Currently, researchers are investigating auto-scaling techniques that can efficiently manage the allocation of different resources. This involves selecting the most suitable resource size and type depending on workload features and scaling and implementing scaling policies for different types of resources. The work in [63] is a promising research direction that needs further exploration. The authors proposed on-demand provisioning of computing resource (OPCR) algorithms that are used to handle and process several applications, which leads to cost savings. However, their proposed algorithm was designed and tested using only simulations. Hence, real-time applications should be implemented to determine accuracy and address challenges in practical environments.

### 7.7. Multi-Cloud Auto-Scaling

Recently, researchers have focused on auto-scaling methods that support hybrid cloud architectures and multi-cloud providers. With a significant increase in the adoption of multi-cloud systems, there is a growing need for auto-scaling algorithms. In single-cloud providers, the scaling decisions specifically made depend on characteristics such as memory utilization and CPU usage. However, in multi-cloud environments, auto-scaling techniques can operate across multiple cloud providers. This ensures optimal resource allocation considering various factors, such as cost, performance, and data locality. Therefore, researchers have identified various areas to enable auto-scaling across multiple cloud environments. One of these important research areas is the investigation of different techniques for efficient workload migration. Moving workloads across several cloud providers can be a challenge; thus, suitable techniques can be implemented to address these challenges. Another research area that researchers should investigate is intelligent workload placement algorithms. These algorithms enhance resource allocation while increasing the efficiency of auto-scaling between different cloud providers.

### 7.8. Intelligent Resource Scaling

Intelligent resource scaling (IRS) is a potential research area that requires further investigation. IRS means the dynamic allocation of computing resources to meet the system’s evolving needs, including auto-scaling resources up or down depending on specific factors. A potential research direction discussed [64], they proposed an IRS for efficient digital twin (DT) simulation. The IRS maintains constant monitoring of the container’s workloads and forecasts resource demands using the DLinear to react to future workloads. Therefore, the IRS still needs further improvements to enhance the reliability and efficiency of the DT simulation. Also, the research area as in [65], needs further improvement and evaluation. The authors mentioned that they plan to investigate online job requirements, which is an estimation technique that provides a highly dynamic scheduling environment. These studies offer promising research directions for intelligent resource scaling, and their focus is on providing different solutions to modern cloud-based applications.

### 7.9. Enhancing Security in Auto-Scaling

Security and privacy are important considerations in auto-scaling systems. Auto-scaling has been widely adopted in different domains; thus, ensuring the security of auto-scaling mechanisms is critical. In [26], the authors discussed key elements of data security, focusing on various aspects of cloud computing. Advanced techniques that improve the security of auto-scaling applications are promising research directions. Furthermore, during the auto-scaling process, privacy-preserving algorithms can be implemented to manage sensitive data. Another important aspect of auto-scaling is adopting homomorphic encryption techniques, which ensures that unauthorized users cannot access sensitive data. Exploring further solutions for these techniques will enhance the security and privacy of auto-scaling systems. Integrating ML technologies is another important research direction for auto-scaling systems. ML algorithms can be trained to detect abnormal behaviors and patterns within auto-scaling applications. Further, it can raise alerts when detecting suspicious activities or indicating security breaches. In [66], researchers presented a malicious traffic detection framework using an unsupervised DL model, achieving a significant 100% accuracy in malicious traffic detection. Table 4 summarizes the promising future research directions provided in this section.

## 8. Conclusions

In this paper, a literature survey of auto-scaling techniques was conducted, and we outlined the main challenges of auto-scaling. Then, we presented several promising future research directions based on the challenges. The different types of auto-scaling techniques were presented and discussed, including reactive methods (threshold rules, queuing theory) and proactive methods (RL, fuzzy learning, ML, and time series analysis). We discussed existing real-world applications in cloud computing and the key challenges related to these use cases. Additionally, we explained the fundamental concepts of cloud computing, auto-scaling, horizontal scaling, and vertical scaling. In future work, we will explore specific areas in auto-scaling. For example, we plan to focus more on resource allocation algorithms.

## Figures and Tables

**Table 1:** List of Acronyms and Their Definition.

Acronym	Definition
RL	Reinforcement learning
SSCAS	Self-Adaptive cloud auto-scaling system
IaaS	Infrastructure as a Service
AWS	Amazon Web Services
QoS	Quality of service
MCS	Mission Critical Services
MDP	Markov decision process
ML	Machine learning
DNN	Deep neural network
CNFs	Containerized network functions
VNFs	Virtualized network functions
VMs	Virtual machines
SLAs	Service level agreements
HDFS	Hadoop’s Distributed File System
Telco	Telecommunications
CEAS	Cost-Effective Auto-Scaling
SVM	Support Vector Machine
OPCR	On-demand provisioning of computing resource
IRS	Intelligent resource scaling
IoT	Internet of things
DT	Digital twin

**Table 2:** Comparison with previous related papers.

Paper	Objective(s)	Advantages	Limitations
**Fourati et al. [12]**	To investigate and analyze existing microservice-based applications that use containers.	Discussed the challenges faced by present solutions and provided future development recommendations. Highlights the strengths and shortcomings of existing microservice-aware auto-scalers.	This study focused on resource allocation in microservice-based applications with containers and did not address other auto-scaling issues, such as cost optimization and performance.
**Tran et al. [13]**	To provide an overview of existing container auto-scaling techniques in Kubernetes.	Analyzed auto-scaling methods, such as horizontal, vertical, and hybrid scaling. Identifies the implementation of machine learning for predictive techniques.	This survey does not cover all auto-scaling techniques; it focuses on auto-scaling in Kubernetes. Lack of practical applications and examples.
**Singh et al. [14]**	To conduct a methodological survey on auto-scaling techniques for web applications. To identify and summarize the challenges and existing studies related to web application auto-scaling.	Describes the key challenges facing multi-tier web applications in cloud computing. Provides taxonomy to help readers understand different aspects of web application auto-scaling.	This survey covered only web application techniques, excluding other aspects of the scalability of cloud computing. In addition, the review literature excludes recent papers.
**Qu et al. [15]**	To review the challenges related to auto-scaling web applications in cloud computing.	The survey covered the weaknesses and strengths of existing auto-scaling techniques deployed by service and infrastructure providers.	Did not cover recent studies conducted after 2019, and the paper focused only on auto-scaling web applications.
**Garí et al. [16]**	To review studies on reinforcement learning (RL) and propose taxonomies to compare RL-based auto-scaling approaches.	The survey covered major proposals for RL-based auto-scaling in cloud infrastructure. In addition, this paper discusses related issues and highlights future research directions.	They focused on RL-based auto-scaling and excluded other aspects of auto-scaling in cloud computing. They excluded real-world applications and domains.
**Garí et al. [17]**	To summarize existing studies and approaches that used RL in the cloud for workflow auto-scaling applications.	Covers most of the studies in the field and highlight the limitations and gaps of other surveys. The survey also covered the opportunities for advancing learning RL-based workflow in the cloud.	Lack of real-world applications and examples. The authors focused particularly on RL-based workflow in the cloud.
**Dogani et al. [18]**	To review auto-scaling techniques for container-based virtualization in cloud, edge, and fog computing.	This review provides a comprehensive understanding of challenges related to auto-scalers for container-based applications.	They focused specifically on auto-scaling techniques for container-based virtualization, excluding other auto-scaling techniques.
**Chen et al. [19]**	To analyze existing studies on Self-Aware and Self-Adaptive cloud auto-scaling systems (SSCAS)	Presented a detailed analysis of open challenges in SSCAS approaches. The paper identified promising directions for further advancement of SSCAS.	The survey excludes auto-scaling concepts and techniques. This study focused on the SSCAS but did not cover other aspects of cloud computing.
**Rabiu et al. [20]**	To determine the current issues of load balancing and understand the challenges related to cloud-based container microservices.	Adds valuable insight into existing challenges faced in load balancing and auto-scaling.	Lack of auto-scaling techniques information. The survey focused on auto-scaling and load balancing in cloud-based container microservices.
**Ali Khan et al. [21]**	To investigate and compare the two techniques of auto-scaling: fixed threshold-based and adaptive threshold-based.	Provides comprehensive insights into fixed threshold-based and adaptive threshold-based techniques and how they can optimize resource allocation.	The paper does not cover challenges related to auto-scaling techniques. They do not address possible solutions and research directions for auto-scaling.
**Our work**	To explore the concept of auto-scaling in cloud computing, algorithms predominantly employed in auto-scaling, to address the challenges associated with auto-scaling in cloud computing and present potential solutions.	Covers recent studies in auto-scaling techniques. Provides research directions that address future challenges. Focuses on auto-scaling techniques (reactive, proactive) and provides detailed discussion about each method.	-

**Table 3:** Auto-scaling techniques used by various cloud providers.

Cloud Providers	Auto-Scaling Feature	Auto-Scaling Supported (Yes/No)
AMAZON	Automatically scales number of EC2 instances for different applications.	Yes
WINDOWS AZURE	Provides auto-scaling feature manually based on the applications.	Yes
GOOGLE APP	Owns auto-scaling technology.	Yes
ENGINE	Google applications.	Yes
GOGRID	Supports auto-scaling technique in programmatic way and does not implement it.	Yes/No
FLEXISCALE	Provides auto-scaling mechanism with high performance and availability.	Yes
ANEKA	Application management service through cloud peer service.	Yes
NIMBUS	Open-source cloud provided by resource manager and Python modules.	Yes
EUCALYPTUS	Open-source cloud which provides wrapper service for various applications.	Yes
OPEN NEBULA	Open-source cloud which provides OpenNebula Service Management Project.	Yes

**Table 4:** Summary of future research directions in auto-scaling.

Issue	Possible Research Direction
Energy efficiency	Energy consumption is a major concern in auto-scaling and some of the existing work tried to minimize energy utilization.
Workload prediction	Using algorithms such as support vector machines, deep learning, and time series analysis enhanced the accuracy.
Cost optimization	Spot instances have emerged recently as a promising solution introduced by AWS that minimizes the cost of resources
Combining vertical and horizontal scaling	Hybrid scaling provides more flexible solutions to enhance resource usage.
Real-time and proactive auto-scaling	Real-time auto-scaling minimizes the response time and enhances system performance.
Heterogeneous resource provisioning	Researchers are investigating auto-scaling techniques that efficiently manage the allocation of different resources.
Multi-cloud auto-scaling	With the significant increase in the adopting of multi-cloud platforms, there is a growing need for auto-scaling algorithms.
Intelligent resource scaling	IRS is the dynamic allocation of computing resources to meet the system’s evolving needs.
Enhancing security in auto-scaling	Developing advanced techniques that improve the security of auto-scaling applications is a promising research direction.

## Data Availability

Data are contained within the article.

## References

[1] Goel P.K., Gulati S., Singh A., Tyagi A., Komal K., Mahur L.S. (2024). Energy-Efficient Block-Chain Solutions for Edge and Cloud Computing Infrastructures. Proceedings of the 2024 2nd International Conference on Disruptive Technologies (ICDT).

[2] Ismahene N.W., Souheila B., Nacereddine Z. (2020). An Auto Scaling Energy Efficient Approach in Apache Hadoop. Proceedings of the 2020 International Conference on Advanced Aspects of Software Engineering (ICAASE).

[3] Eljak H., Ibrahim A.O., Saeed F., Hashem I.A.T., Abdelmaboud A., Syed H.J., Abulfaraj A.W., Ismail M.A., Elsafi A. (2023). E-learning based Cloud Computing Environment: A Systematic Review, Challenges, and Opportunities. IEEE Access.

[4] Fathullah M.A., Subbarao A., Muthaiyah S., Taralunga D. (2024). Risk Classes of Cloud Computing Project in Healthcare: A Review of Technical Report and Standards. J. Adv. Res. Appl. Sci. Eng. Technol..

[5] Bodemer O. (2024). Revolutionizing Finance: The Impact of AI and Cloud Computing in the Banking Sector. TechRxiv.

[6] Kiatipis A., Xanthopoulos A. (2024). Cloud Usage for Manufacturing: Challenges and Opportunities. Procedia Comput. Sci..

[7] Sao Cao D., Nguyen D.T., Nguyen X.C., Nguyen H.B., Lang K.T., Dao N.L., Pham T.T., Cao N.S., Chu D.H., Nguyen P.H. (2023). Elastic auto-scaling architecture in telco cloud. Proceedings of the 2023 25th International Conference on Advanced Communication Technology (ICACT).

[8] Boujelben Y., Fourati H. (2024). A distributed auction-based algorithm for virtual machine placement in multiplayer cloud gaming infrastructures. Int. J. Cloud Comput..

[9] Singh P.D., Singh K.D. (2024). Interdisciplinary Approaches: Fog/Cloud Computing and IoT for AI and Robotics Integration. EAI Endorsed Trans. AI Robot..

[10] Heidari S., Buyya R. (2019). A cost-efficient auto-scaling algorithm for large-scale graph processing in cloud environments with heterogeneous resources. IEEE Trans. Softw. Eng..

[11] Simic V., Stojanovic B., Ivanovic M. (2019). Optimizing the performance of optimization in the cloud environment–An intelligent auto-scaling approach. Future Gener. Comput. Syst..

[12] Fourati M.H., Marzouk S., Jmaiel M. (2023). Towards Microservices-Aware Autoscaling: A Review. Proceedings of the 2023 IEEE Symposium on Computers and Communications (ISCC).

[13] Tran M.N., Vu D.D., Kim Y. (2022). A survey of autoscaling in kubernetes. Proceedings of the 2022 Thirteenth International Conference on Ubiquitous and Future Networks (ICUFN).

[14] Singh P., Gupta P., Jyoti K., Nayyar A. (2019). Research on auto-scaling of web applications in cloud: Survey, trends and future directions. Scalable Comput. Pract. Exp..

[15] Qu C., Calheiros R.N., Buyya R. (2018). Auto-scaling web applications in clouds: A taxonomy and survey. ACM Comput. Surv. (CSUR).

[16] Garí Y., Monge D.A., Pacini E., Mateos C., Garino C.G. (2021). Reinforcement learning-based application autoscaling in the cloud: A survey. Eng. Appl. Artif. Intell..

[17] Gari Y., Monge D.A., Pacini E., Mateos C., Garino C.G. (2020). Reinforcement learning-based autoscaling of workflows in the cloud: A survey. arXiv.

[18] Dogani J., Namvar R., Khunjush F. (2023). Auto-scaling techniques in container-based cloud and edge/fog computing: Taxonomy and survey. Comput. Commun..

[19] Chen T., Bahsoon R., Yao X. (2018). A survey and taxonomy of self-aware and self-adaptive cloud autoscaling systems. ACM Comput. Surv. (CSUR).

[20] Rabiu S., Yong C.H., Mohamad S.M.S. (2022). A cloud-based container microservices: A review on load-balancing and auto-scaling issues. Int. J. Data Sci..

[21] Khan A.A., Vidhyadhari C.H., Kumar S. (2024). A review on fixed threshold based and adaptive threshold based auto-scaling techniques in cloud computing. MATEC Web Conf..

[22] Hung C.L., Hu Y.C., Li K.C. (2012). Auto-scaling model for cloud computing system. Int. J. Hybrid Inf. Technol..

[23] Solino A., Batista T., Cavalcante E. (2023). Decision-Making Support to Auto-scale Smart City Platform Infrastructures. Proceedings of the 2023 18th Iberian Conference on Information Systems and Technologies (CISTI).

[24] Jannapureddy R., Vien Q.T., Shah P., Trestian R. (2019). An auto-scaling framework for analyzing big data in the cloud environment. Appl. Sci..

[25] Alipour H., Liu Y., Hamou-Lhadj A. (2014). Analyzing auto-scaling issues in cloud environments. CASCON.

[26] Atadoga A., Umoga U.J., Lottu O.A., Sodiya E.O. (2024). Evaluating the impact of cloud computing on accounting firms: A review of efficiency, scalability, and data security. Glob. J. Eng. Technol. Adv..

[27] Mullapudi M., Munjala M.B., Kulkarni C. Designing a Resilient Parallel Distributed Task Infrastructure for Large-Scale Data Processing. https://www.researchgate.net/profile/Mahidhar-Mullapudi/publication/378438900_Designing_a_Resilient_Parallel_Distributed_Task_Infrastructure_for_Large-scale_Data_Processing/links/65d96d94adc608480ae7fa04/Designing-a-Resilient-Parallel-Distributed-Task-Infrastructure-for-Large-scale-Data-Processing.pdf.

[28] Singh S.T., Tiwari M., Dhar A.S. (2023). Machine Learning based Workload Prediction for Auto-scaling Cloud Applications. Proceedings of the 2022 OPJU International Technology Conference on Emerging Technologies for Sustainable Development (OTCON).

[29] Ashalatha R., Agarkhed J. (2015). Evaluation of auto scaling and load balancing features in cloud. Int. J. Comput. Appl..

[30] Taha M.B., Sanjalawe Y., Al-Daraiseh A., Fraihat S., Al-E’mari S.R. (2024). Proactive Auto-Scaling for Service Function Chains in Cloud Computing based on Deep Learning. IEEE Access.

[31] Ahamed Z., Khemakhem M., Eassa F., Alsolami F., Al-Ghamdi A.S.A.M. (2023). Technical study of deep learning in cloud computing for accurate workload prediction. Electronics.

[32] Pereira P., Araujo J., Maciel P. (2019). A hybrid mechanism of horizontal auto-scaling based on thresholds and time series. Proceedings of the 2019 IEEE International Conference on Systems, Man and Cybernetics (SMC).

[33] Quattrocchi G., Incerto E., Pinciroli R., Trubiani C., Baresi L. (2024). Autoscaling Solutions for Cloud Applications under Dynamic Workloads. IEEE Trans. Serv. Comput..

[34] Hu Y., Deng B., Peng F. (2016). Autoscaling prediction models for cloud resource provisioning. Proceedings of the 2016 2nd IEEE International Conference on Computer and Communications (ICCC).

[35] Malla P.A., Sheikh S., Shahid M., Mushtaq S.U. (2024). Energy-efficient sender-initiated threshold-based load balancing (e-STLB) in cloud computing environment. Concurr. Comput. Pract. Exp..

[36] Tournaire T., Castel-Taleb H., Hyon E. (2023). Efficient Computation of Optimal Thresholds in Cloud Auto-scaling Systems. ACM Trans. Model. Perform. Eval. Comput. Syst..

[37] Kushchazli A., Safargalieva A., Kochetkova I., Gorshenin A. (2024). Queuing Model with Customer Class Movement across Server Groups for Analyzing Virtual Machine Migration in Cloud Computing. Mathematics.

[38] Heimerson A., Eker J., Årzén K.E. (2022). A Proactive Cloud Application Auto-Scaler using Reinforcement Learning. Proceedings of the 2022 IEEE/ACM 15th International Conference on Utility and Cloud Computing (UCC).

[39] Khaleq A.A., Ra I. (2021). Intelligent autoscaling of microservices in the cloud for real-time applications. IEEE Access.

[40] Joyce J.E., Sebastian S. (2022). Reinforcement learning based autoscaling for kafka-centric microservices in kubernetes. Proceedings of the 2022 IEEE 4th PhD Colloquium on Emerging Domain Innovation and Technology for Society (PhD EDITS).

[41] Alnawayseh S.E., Muhammad M.H.G., Hassan Z., Fatima M., Aslam M.S., Ibrahim A., Ateeq K. (2023). Resource Provisioning in Cloud Computing using Fuzzy Logic Control System: An adaptive approach. Proceedings of the 2023 International Conference on Business Analytics for Technology and Security (ICBATS).

[42] Mahapatra A., Majhi S.K., Mishra K., Pradhan R., Rao D.C., Panda S.K. (2024). An energy-aware task offloading and load balancing for latency-sensitive IoT applications in the Fog-Cloud continuum. IEEE Access.

[43] Abdulazeez D.H., Askar S.K. (2024). A Novel Offloading Mechanism Leveraging Fuzzy Logic and Deep Reinforcement Learning to Improve IoT Application Performance in a Three-Layer Architecture within the Fog-Cloud Environment. IEEE Access.

[44] Luan S., Shen H. (2024). Minimize Resource Cost for Containerized Microservices Under SLO via ML-Enhanced Layered Queueing Network Optimization. Proceedings of the 2024 14th International Conference on Cloud Computing, Data Science & Engineering (Confluence).

[45] Jensen A. (2024). AI-Driven DevOps: Enhancing Automation with Machine Learning in AWS. Integr. J. Sci. Technol..

[46] Chhabra M., Arora G. (2023). The Impact of Machine Learning (ML) Driven Algorithm Ranking and Visualization on Task Scheduling in Cloud Computing. Proceedings of the 2023 3rd International Conference on Advancement in Electronics & Communication Engineering (AECE).

[47] Yang R., Ouyang X., Chen Y., Townend P., Xu J. (2018). Intelligent resource scheduling at scale: A machine learning perspective. Proceedings of the 2018 IEEE Symposium on Service-Oriented System Engineering (SOSE).

[48] Spantideas S., Giannopoulos A., Cambeiro M.A., Trullols-Cruces O., Atxutegi E., Trakadas P. (2023). Intelligent Mission Critical Services over Beyond 5G Networks: Control Loop and Proactive Overload Detection. Proceedings of the 2023 International Conference on Smart Applications, Communications and Networking (SmartNets).

[49] Pfister B.J., Scheinert D., Geldenhuys M.K., Kao O. (2024). Daedalus: Self-Adaptive Horizontal Autoscaling for Resource Efficiency of Distributed Stream Processing Systems. arXiv.

[50] Gkontzis A.F., Kotsiantis S., Feretzakis G., Verykios V.S. (2024). Temporal Dynamics of Citizen-Reported Urban Challenges: A Comprehensive Time Series Analysis. Big Data Cogn. Comput..

[51] Westergaard G., Erden U., Mateo O.A., Lampo S.M., Akinci T.C., Topsakal O. (2024). Time Series Forecasting Utilizing Automated Machine Learning (AutoML): A Comparative Analysis Study on Diverse Datasets. Information.

[52] Muccini H., Vaidhyanathan K. (2019). A machine learning-driven approach for proactive decision making in adaptive architectures. Proceedings of the 2019 IEEE international conference on software architecture companion (ICSA-C).

[53] Ahn Y.W., Cheng A.M., Baek J., Jo M., Chen H.H. (2013). An auto-scaling mechanism for virtual resources to support mobile, pervasive, real-time healthcare applications in cloud computing. IEEE Netw..

[54] Dougherty B., White J., Schmidt D.C. (2012). Model-driven auto-scaling of green cloud computing infrastructure. Future Gener. Comput. Syst..

[55] Grimson M., Almeida R., Shi Q., Bai Y., Angarita H., Pacheco F.S., Schmitt R., Flecker A., Gomes C.P. Scaling Up Pareto Optimization for Tree Structures with Affine Transformations: Evaluating Hybrid Floating Solar-Hydropower Systems in the Amazon. Proceedings of the AAAI Conference on Artificial Intelligence.

[56] Jayanetti A., Halgamuge S., Buyya R. (2024). Multi-Agent Deep Reinforcement Learning Framework for Renewable Energy-Aware Workflow Scheduling on Distributed Cloud Data Centers. IEEE Trans. Parallel Distrib. Syst..

[57] Baynum A., Hao W. (2024). Exploring the Impact of Cloud Computing and Edge Computing on Resource Consumption for Mobile Devices with Generative Artificial Intelligence APIs. Proceedings of the 2024 IEEE 14th Annual Computing and Communication Workshop and Conference (CCWC).

[58] Shi T., Ma H., Chen G., Hartmann S. (2023). Auto-Scaling Containerized Applications in Geo-Distributed Clouds. IEEE Trans. Serv. Comput..

[59] Meng C., Tong H., Wu T., Pan M., Yu Y. (2024). Multi-Level ML Based Burst-Aware Autoscaling for SLO Assurance and Cost Efficiency. arXiv.

[60] Choonhaklai P., Chantrapornchai C. (2023). Two Autoscaling Approaches on Kubernetes Clusters Against Data Streaming Applications. Proceedings of the 2023 International Technical Conference on Circuits/Systems, Computers, and Communications (ITC-CSCC).

[61] Vu D.D., Tran M.N., Kim Y. (2022). Predictive hybrid autoscaling for containerized applications. IEEE Access.

[62] Ju L., Singh P., Toor S. Proactive autoscaling for edge computing systems with kubernetes. Proceedings of the IEEE/ACM International Conference on Utility and Cloud Computing Companion.

[63] Wang Y., Li Y., Guo J., Fan Y., Chen L., Zhang B., Wang W., Zhao Y., Zhang J. (2023). On-demand provisioning of computing resources in computing power network with mixed CPU and GPU. Proceedings of the 2023 21st International Conference on Optical Communications and Networks (ICOCN).

[64] Jeon J., Jeong B., Jeong Y.S. (2023). Intelligent resource scaling for container based digital twin simulation of consumer electronics. IEEE Trans. Consum. Electron..

[65] Gu R., Chen Y., Liu S., Dai H., Chen G., Zhang K., Che Y., Huang Y. (2021). Liquid: Intelligent resource estimation and network-efficient scheduling for deep learning jobs on distributed GPU clusters. IEEE Trans. Parallel Distrib. Syst..

[66] Hwang R.H., Peng M.C., Huang C.W., Lin P.C., Nguyen V.L. (2020). An unsupervised deep learning model for early network traffic anomaly detection. IEEE Access.

